# Effects of RNA Binding Proteins on the Prognosis and Malignant Progression in Prostate Cancer

**DOI:** 10.3389/fgene.2020.591667

**Published:** 2020-10-20

**Authors:** Xiaoliang Hua, Shengdong Ge, Juan Chen, Li Zhang, Sheng Tai, Chaozhao Liang

**Affiliations:** ^1^Department of Urology, The First Affiliated Hospital of Anhui Medical University, Hefei, China; ^2^Anhui Province Key Laboratory of Genitourinary Diseases, Anhui Medical University, Hefei, China; ^3^The Institute of Urology, Anhui Medical University, Hefei, China; ^4^The Ministry of Education Key Laboratory of Clinical Diagnostics, School of Laboratory Medicine, Chongqing Medical University, Chongqing, China

**Keywords:** prostate cancer, RNA binding protein, bioinformatics, biomarker, prognostic model

## Abstract

Prostate cancer (PCa) is a common lethal malignancy in men. RNA binding proteins (RBPs) have been proven to regulate the biological processes of various tumors, but their roles in PCa remain less defined. In the present study, we used bioinformatics analysis to identify RBP genes with prognostic and diagnostic values. A total of 59 differentially expressed RBPs in PCa were obtained, comprising 28 upregulated and 31 downregulated RBP genes, which may play important roles in PCa. Functional enrichment analyses showed that these RBPs were mainly involved in mRNA processing, RNA splicing, and regulation of RNA splicing. Additionally, we identified nine RBP genes (EXO1, PABPC1L, REXO2, MBNL2, MSI1, CTU1, MAEL, YBX2, and ESRP2) and their prognostic values by a protein–protein interaction network and Cox regression analyses. The expression of these nine RBPs was validated using immunohistochemical staining between the tumor and normal samples. Further, the associations between the expression of these nine RBPs and pathological T staging, Gleason score, and lymph node metastasis were evaluated. Moreover, these nine RBP genes showed good diagnostic values and could categorize the PCa patients into two clusters with different malignant phenotypes. Finally, we constructed a prognostic model based on these nine RBP genes and validated them using three external datasets. The model showed good efficiency in predicting patient survival and was independent of other clinical factors. Therefore, our model could be used as a supplement for clinical factors to predict patient prognosis and thereby improve patient survival.

## Introduction

Prostate cancer (PCa), one of the most common and lethal neoplasms in the urologic system, results in approximately 260,000 annual deaths in men worldwide (Siegel et al., 2020). For the past few decades, the incidence rate of PCa has been constantly rising in developing countries and posing a great burden on public health systems (Zhu et al., 2015). At present, the main monitoring indicators of PCa include serum prostate-specific antigen (PSA) levels and pathological stage identification. Therefore, new biomarkers are needed to aid in the diagnosis and timely treatment of PCa. With advances in medical research, the disease-free survival of PCa patients has improved. However, approximately 30% of PCa patients experience recurrence and metastasis after undergoing surgical resection (Tomita et al., 2020). While androgen deprivation therapy is an effective therapeutic method employed in the initial stage of treatment, many PCa patients eventually develop aggressive castration-resistant PCa (CRPC; Graham et al., 2008; Wong et al., 2014). Therefore, the identification of valuable molecular markers and construction of a more effective and specific stratification model are of great significance to guide clinical treatment and improve the prognosis and diagnosis of PCa patients.

Recently, the functions of RNA-binding proteins (RBPs) have been widely studied. An RBP interacts with different classes of target RNA to form ribonucleoprotein complexes and regulates gene expression through RNA processing at the posttranscriptional level (Gerstberger et al., 2014a). The RBPs are abundantly expressed in cells and are involved in nearly every aspect of biological processes, including RNA stability, splicing, modification, transport, location, and translation (Gerstberger et al., 2014b; Perron et al., 2018). Hence, RBPs are critical for the stabilization and development of cells and organisms. The dysregulation of RBPs leads to an aberrant gene expression in cells, which may ultimately result in a disease. Moreover, previous studies have indicated that RBPs play a significant role in the initiation and progression of PCa; for instance, *TDRD1*, an ERG target gene, can promote the occurrence and development of PCa (Xiao et al., 2016), and PCBP1 could increase the tumorigenicity and metastasis of PCa by inhibiting the expression of mitogen-activated protein kinase 1 (Zhang et al., 2018). Further, multiple RBPs can regulate the androgen receptor (AR) pathway to influence PCa neoplasia and progression; for instance, HNRNPL is aberrantly expressed in PCa and regulates the alternative splicing of many types of RNA, including those encoding the AR, to influence the progression of PCa (Fei et al., 2017). In addition, PSF could induce the dysregulation of various spliceosome genes to promote the amplification and splicing of the AR in advanced PCa (Takayama et al., 2017). Further, Sam68 could enhance the expression of the AR and modulate the transcription function of the AR splice variant AR-V7, which drives the progression of CRPC (Stockley et al., 2015). Finally, the expression of Musashi2 is positively correlated with tumor grades and drives PCa progression by binding to the 3′-untranslated region to stabilize the AR (Zhao et al., 2020). However, the molecular functions of most RBPs involved in the tumorigenesis and progression of PCa have not been thoroughly studied. Therefore, a systematic study of the RBPs will not only help in discovering their potential values in PCa but also contribute in identifying specific and effective diagnostic and prognostic biomarkers.

Hence, we used comprehensive bioinformatic methods to identify potential biomarkers for PCa patients and constructed an RBP–based risk score model to stratify the patients. We acquired the relevant datasets and clinical information from public databases to screen for the RBP genes. Then, we investigated their prognostic impact in PCa through functional enrichment analyses, protein–protein interaction (PPI) networks, and Cox regression analyses. Finally, we validated our model in external datasets and identified the association of the key RBPs with different clinicopathological factors.

## Materials and Methods

### Dataset Acquisition

**FIGURE 1:**
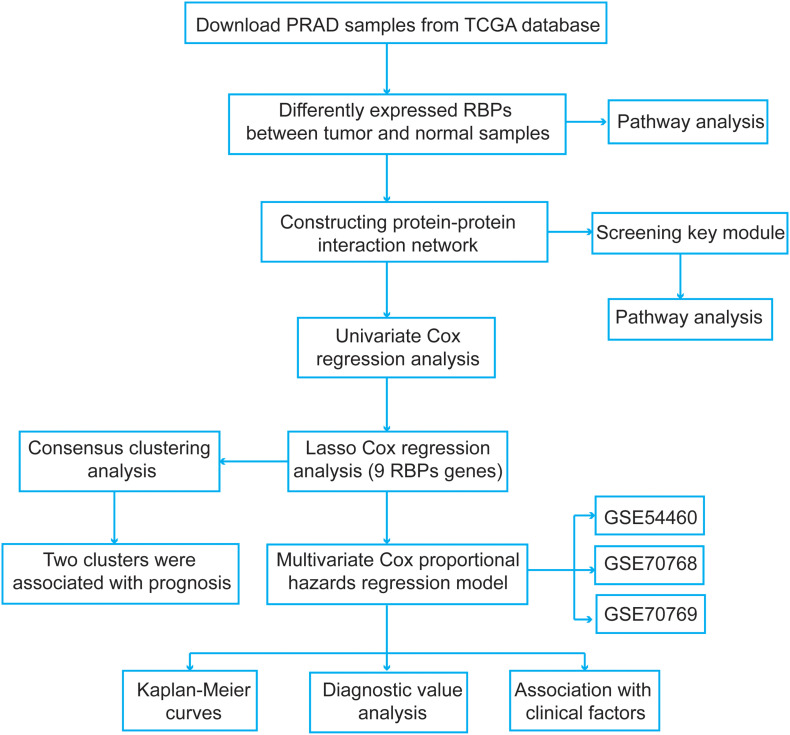
The flowchart of the present study design.

We explored the pivotal roles and prognostic values of RBPs in PCa using an integrated bioinformatics analysis. The flowchart of this study is shown in Figure 1. The datasets were obtained from The Cancer Genome Atlas (TCGA^1^) and the Gene Expression Omnibus (GEO^2^) database. For TCGA dataset, the expression data and clinical information were downloaded using UCSC Xena^3^. Further, the disease-free survival information of the PCa patients was obtained from the cBio Cancer Genomics Portal^4^. A total of 52 normal samples and 498 PCa samples were obtained. Then, a differential expression analysis was performed between the PCa and normal samples using the “limma” package^5^ of R with the following criteria: false discovery rate (FDR) < 0.05 and |fold change| > 2. For the expression data in TCGA dataset, the data were log2(*x* + 1) transformed for normalization by the “RNA-Seq by Expectation-Maximization” package^6^. To select genes with prognostic values and establish a risk score model, PCa samples were screened based on following criteria: (1) repeated tumor samples in the same patient were removed, and (2) patients with unknown disease-free survival status and follow-up information were excluded. Finally, 491 PCa samples meeting the inclusion criteria were included.

Next, the normalized microarray datasets, including GSE54460, GSE70768, and GSE70769, were directly downloaded from the GEO database. For the GSE54460 dataset, the expression data were measured by fragments per kilobase per million values. The GSE70768 and GSE70769 datasets were produced using the Illumina HumanHT-12 V4.0 expression BeadChip platform, and the probes were annotated using the corresponding “illuminaHumanv4.db” R package. The data of duplicate genes were averaged. The expression data in these two datasets were log2 transformed and quantile normalized. The GSE54460 dataset included 90 PCa samples and corresponding disease-free survival information. The GSE70768 and GSE70769 datasets included 111 and 92 PCa samples, respectively. These three datasets were used to validate our model. Finally, we obtained a list of RBPs from a previous study (Gerstberger et al., 2014b) and included a total of 1,524 RBPs in our study.

### Functional Enrichment Analyses

We performed Gene Ontology (GO) and Kyoto Encyclopedia of Genes and Genomes (KEGG) enrichment analyses of the differentially expressed RBPs using the Database for Annotation, Visualization and Integrated Discovery^7^. We identified enriched terms for biological processes, cellular components, and molecular functions; a *P*-value <0.05 was set as the cutoff value. Moreover, a Gene Set Enrichment Analysis (GSEA) was performed to ascertain the molecular functional mechanisms. We selected the “h.all.v7.1.symbols.gmt” file as the reference gene set file and set FDR < 0.25 and normalized *P*-value <0.05 as the threshold values.

### Construction of a Protein–Protein Interaction (PPI) Network and Screening for the Key Modules

We submitted the differentially expressed RBP genes in the Search Tool for the Retrieval of Interacting Genes database (STRING)^8^ to construct a PPI network and further explore the potential molecular functions of these RBPs in tumorigenesis and progression of PCa. Subsequently, we extracted and visualized genes with an interaction score of 0.4 using Cytoscape v3.7.1 software^9^. Finally, we screened the key modules from the PPI network with a *k*-core value of 4 using the Molecular Complex Detection (MCODE) plugin in Cytoscape.

### Identification and Validation of the Survival-Related RBPs

To identify survival-related RBP genes, we performed a univariate Cox regression analysis for the differentially expressed RBPs. Next, we used the least absolute shrinkage and selection operator (lasso) Cox regression analysis (Tibshirani, 1997) to screen the most significant prognostic RBPs of PCa using the “survival” and “glmnet” R package^10^. The optimal values of penalty parameters (lambda value) were determined by 10-fold cross-validation. Then, the Kaplan–Meier curves were plotted and log-rank tests were performed to verify the prognostic values of these survival-related RBP genes. A *P*-value <0.05 was set as the cutoff value. We further validated the expression levels of these RBP genes in the Human Protein Atlas (HPA) database (Uhlen et al., 2017). Then, the receiver operating characteristic (ROC) curves and the areas under the curves (AUCs) were calculated using the “pROC” package^11^ in R to evaluate the diagnostic efficiency of these RBPs (Sing et al., 2005). Furthermore, we utilized the segmentation analysis and “Genomic Identification of Significant Targets in Cancer” algorithm from cBioPortal (GISTIC) (see text footnote 4) to determine the mutation and copy number alteration changes of each survival-related RBP (Gao et al., 2013).

### Consensus Clustering of the Survival-Related RBPs

To further detect the functions and prognostic values of the RBPs in PCa, we performed a consensus clustering to determine the cluster numbers using the “ConsensusClusterPlus” R package^12^ based on the survival-related RBPs (Wu et al., 2017). Next, a principal component analysis (PCA) was used to assess the distribution patterns and confirm the cluster numbers using the “ggplot2” R package.

### Construction of a Prognostic Model

Based on the selected survival-related RBP genes, we performed a multivariate Cox regression analysis to acquire their coefficients. Then, we constructed a prognostic risk score model to stratify the patients. The risk score was calculated using the following formula:

Riskscore=β1×Exp1+β2×Exp2+βi×Expi

where *β* and *Exp* represent the regression coefficients and gene expression levels, respectively. Finally, the Kaplan–Meier and ROC curves were used to evaluate the efficiency of the risk score model.

### Statistical Analyses

We used Pearson’s chi-square test to investigate the differences in the distributions of the clinical information. We performed a *t*-test or Wilcoxon test for two samples and a Kruskal–Wallis test for multiple samples. The univariate and multivariate Cox regression analyses were performed to evaluate the prognostic values of the RBPs. The Kaplan–Meier curves and log-rank tests were used to identify the survival difference. All procedures involved in the present study were conducted using the R software. All statistical results were considered to be significant if the *P*-value is <0.05.

## Results

### Acquisition of the Differentially Expressed RBPs

We obtained 59 differentially expressed RBP genes comprising 28 upregulated and 31 downregulated RBPs (Supplementary Table 1). The functional enrichment analyses of the upregulated differentially expressed RBPs revealed the following enriched terms: “translation and rRNA processing” for biological processes; “nucleolus, cytosolic large ribosomal subunit, and ribosome” for cellular component; and “RNA binding, poly(A) RNA binding, and nucleic acid binding” for molecular function (Supplementary Table 2). In contrast, the downregulated RBPs were primarily enriched in “mRNA processing, RNA splicing, regulation of RNA splicing, and cytidine deamination” for biological processes; “cytoplasm and nucleus” for cellular component; and “nucleotide binding, RNA binding, nucleic acid binding, and poly(A) RNA binding” for molecular function (Supplementary Table 3). In addition, the KEGG pathway analysis revealed that the upregulated RBPs were significantly enriched in “ribosome,” “mRNA surveillance pathway,” “RNA degradation,” and “RNA transport” (Supplementary Table 2).

### PPI Network Construction and Module Screening

**FIGURE 2:**
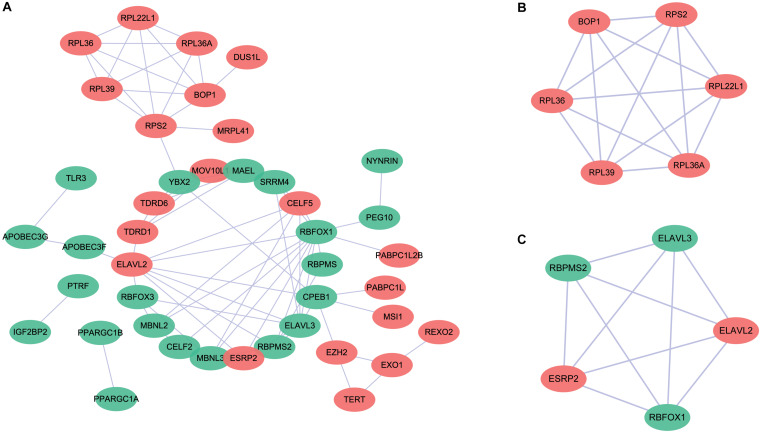
Protein–protein interaction (PPI) network for the differentially expressed RNA binding proteins. **(A)** The upregulated and downregulated genes are shown in red and green circles, respectively. **(B)** Key module 1 in the PPI network. **(C)** Key module 2 in the PPI network.

To further explore the potential molecular functions, we submitted these differentially expressed RBP genes to the STRING database to construct a PPI network (Figure 2). The upregulated and downregulated RBPs are shown in red and green circles, respectively. We obtained a total of 58 PPI nodes and 75 PPI edges with a PPI enrichment *P*-value <1.0e^–16^. The functional enrichment analyses of the PPI network revealed the following enriched terms: “mRNA metabolic process,” “RNA metabolic process,” “RNA process,” “nucleic acid metabolic process,” and “mRNA processing” in biological processes; “RNA binding,” “nucleic acid binding,” “heterocyclic compound binding,” “organic cyclic compound binding,” and “mRNA binding” in molecular function; and “ribonucleoprotein complex,” “cytoplasmic ribonucleoprotein granule,” “cytosolic ribosome,” and “cytosolic large ribosomal subunit” in cellular components. In the KEGG pathway analysis, the enriched terms were “ribosome,” “mRNA surveillance pathway,” and “RNA degradation.” Moreover, two key modules were obtained using the MCODE software (Figures 2B,C). We found that module 1 was mainly enriched in “cytosolic large ribosomal subunit” and “polysomal ribosome” (Figure 2B), while none of the enriched pathways were detected in module 2 (Figure 2C).

### Identification of the RBPs With Prognostic Values

**FIGURE 3:**
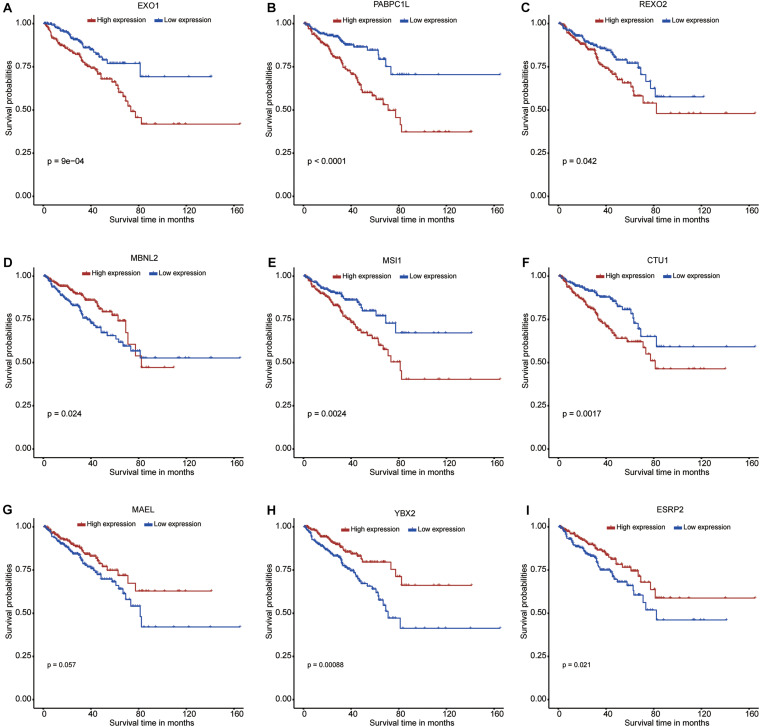
Kaplan–Meier curves for the nine RNA binding proteins in prostate cancer from The Cancer Genome Atlas dataset. **(A)** EXO1; **(B)** PABPC1L; **(C)** REXO2; **(D)** MBNL2; **(E)** MSI1; **(F)** CTU1; **(G)** MAEL; **(H)** YBX2; **(I)** ESRP2.

A total of 58 differentially expressed RBP genes were obtained from the PPI network and used to perform the univariate Cox regression analysis to select survival-related RBPs (Supplementary Table 4). A total of 18 RBPs met the inclusion criterion (*P* < 0.05). These selected RBPs were used to perform the lasso Cox regression analysis to select nine prognostic RBPs (Supplementary Figure 1). The minimum lambda value used in the present study was 0.016. Six RBP genes (*EXO1*, *PABPC1L*, *REXO2*, *MSI1*, *CTU1*, and *ESRP2*) were upregulated, and three RBP genes (*MAEL*, *MBNL2*, and *YBX2*) were downregulated in the PCa samples when compared with normal samples. In addition, the Kaplan–Meier curves further confirmed the prognostic values of these RBPs, including *EXO1*, *PABPC1L*, *REXO2*, *MBNL2*, *MSI1*, *CTU1*, *MAEL*, *YBX2*, and *ESRP2* (Figures 3A–I, respectively).

### Validation of the Nine Survival-Related RBPs

**FIGURE 4:**
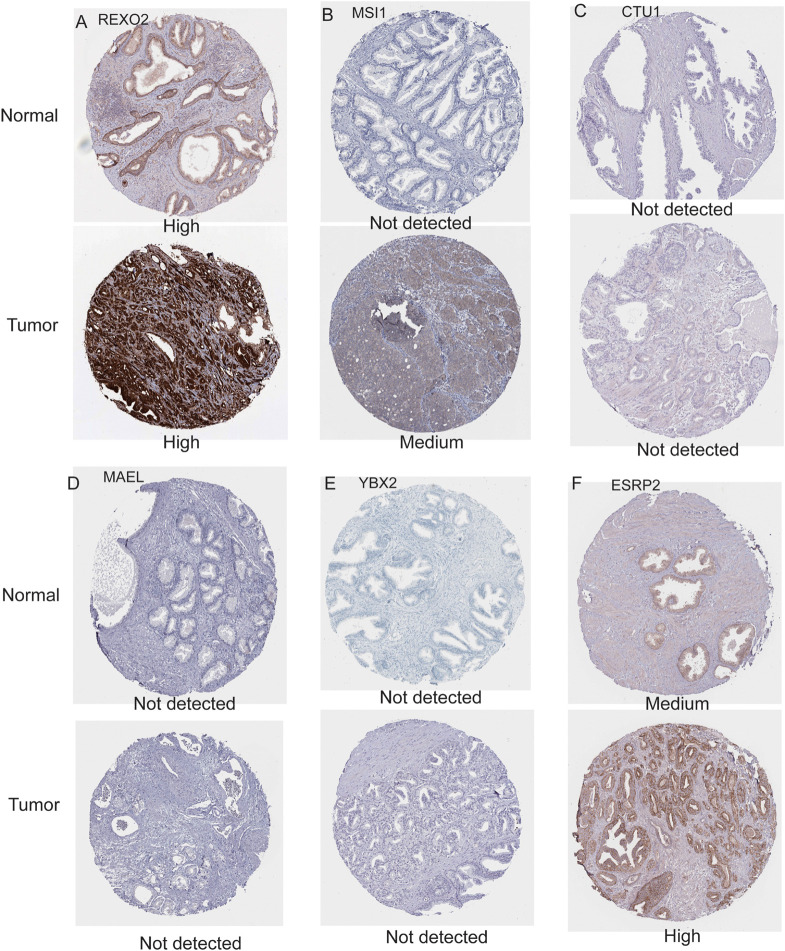
Immunohistochemistry results for RNA binding proteins in normal and prostate cancer tissues from the Human Protein Atlas database. **(A)** REXO2; **(B)** MSI1; **(C)** CTU1; **(D)** MAEL; **(E)** YBX2; **(F)** ESRP2.

**FIGURE 5:**
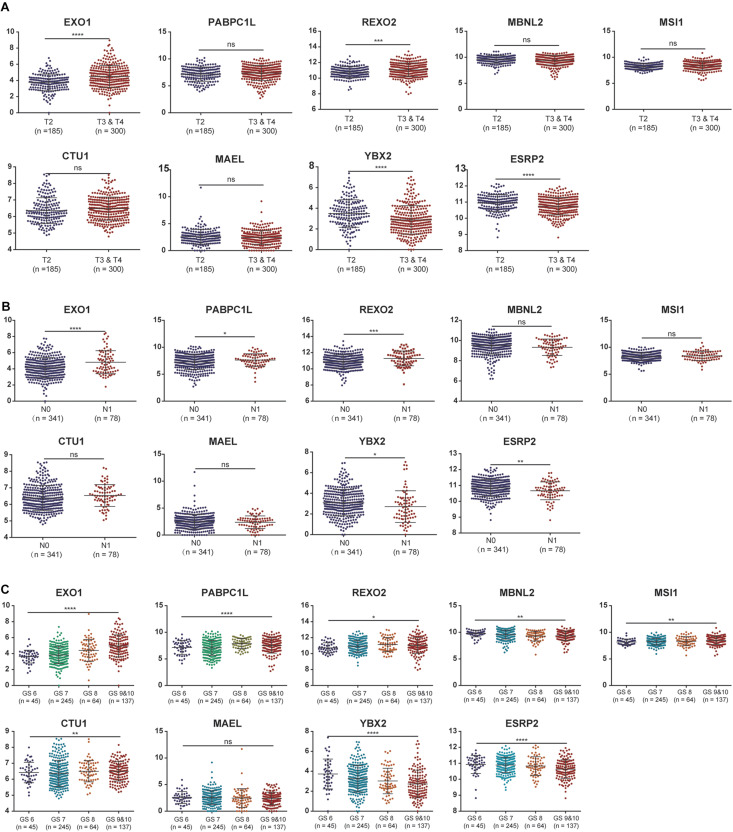
The associations between the expression levels of the nine RNA binding proteins and clinical factors. **(A)** T2 staging vs. T3 and T4 staging; **(B)** N0 staging vs. N1 staging. **(C)** Gleason score for 6, 7, 8, 9, and 10. ns: *P* > 0.05; *P < 0.05; **P < 0.01; ***P < 0.001; ****P < 0.0001. GS represents the Gleason score.

To further evaluate the expression levels of these nine RBPs in PCa, we obtained their immunohistochemical results from the HPA database (EXO1, PABPC1L, and MBNL2 were not tested here). REXO2, MSI1, and ESRP2 had high expression levels in tumors compared with normal tissues, while CTU1, MAEL, and YBX2 were undetermined in both tumor and normal tissues (Figure 4). In addition, we evaluated the diagnostic values of these RBPs to differentiate tumors from normal samples and found that all nine RBP genes showed moderate diagnostic efficiency (Supplementary Figures 2A–I). The mutation and copy number alterations of the RBP genes were determined, and 69 out of 489 (14%) PCa samples were found to be altered (Supplementary Figure 2J); the most frequent alteration was the deep deletion of the *YBX2* gene. Moreover, the mutation frequencies of these nine RBPs were low. Further, the associations between the expression levels of these RBP genes and clinical factors were confirmed in TCGA dataset. We found high expression levels of EXO1 and REXO2 and low expression levels of YBX2 and ESRP2 in samples with high pathological T staging (Figure 5A); high expression levels of EXO1, PABPC1L, and REXO2 and low expression levels of YBX2 and ESRP2 in high pathological grade (Figure 5B); and high expression levels of EXO1, PABPC1L, REXO2, MSI1, and CTU1 and low expression levels of MBNL2, YBX2, and ESRP2 in high Gleason score (Figure 5C). The functional enrichment analysis for these nine RBP genes revealed enrichment in “nucleic acid binding,” “nucleotide binding,” “regulation of RNA splicing,” “RNA binding,” “mRNA surveillance pathway,” and “poly(A) RNA binding.”

### Identification of Two Clusters Using Consensus Clustering

**FIGURE 6:**
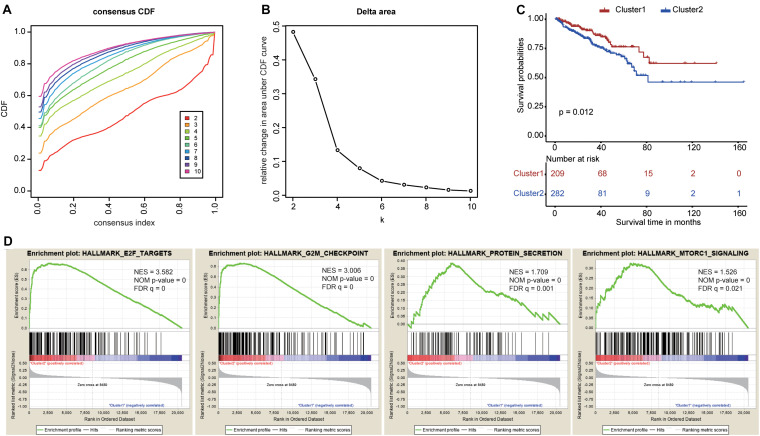
Consensus clustering based on the nine survival-related RNA binding proteins. **(A)** Consensus clustering cumulative distribution function (CDF) for *k* = 2 to *k* = 10. **(B)** The relative change in area under the CDF curve for *k* = 2 to *k* = 10. **(C)** The Kaplan–Meier curve for prostate cancer patients to evaluate disease-free survival. **(D)** The Gene Set Enrichment Analysis showed that several oncogenic pathways were significantly enriched in cluster 2.

To explore the prognostic value of the nine RBPs, we performed a consensus clustering analysis to select cluster numbers based on the similarity of these genes. We found that *k* = 5 seemed to be the most stable value from *k* = 2 to *k* = 10 in TCGA dataset (Figures 6A,B). Then, we performed a PCA analysis to evaluate the reliability of the consensus clustering. The results showed high similarity and overlap when the cluster numbers were three (Supplementary Figures 3C,D), four (Supplementary Figures 3E,F), and five (Supplementary Figures 3G,H). Therefore, we divided the patients into two clusters (Supplementary Figure 3A), and the PCA showed different distributions between these two clusters (Supplementary Figure 3B). The Kaplan–Meier curves showed different prognoses between the two clusters as cluster 2 showed poorer prognosis when compared with cluster 1 (Figure 6C). Finally, the GSEA of these two clusters highlighted several oncogenic pathways significantly enriched in cluster 2 (Figure 6D), including E2F targets [normalized enrichment score (NES) = 3.582, size = 187], G2M checkpoints (NES = 3.006, size = 184), protein secretion (NES = 1.709, size = 95), and mTORC1 signaling (NES = 1.526, size = 192).

### Construction and Validation of a Risk Score Model

**FIGURE 7:**
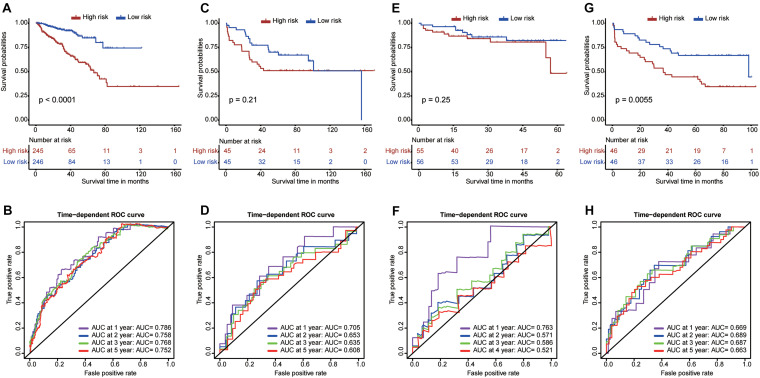
Construction and validation of a risk score model. The Kaplan–Meier curves and time-dependent ROC curves for The Cancer Genome Atlas dataset **(A,B)**, GSE54460 dataset **(C,D)**, GSE70768 dataset **(E,F)**, and GSE70769 dataset **(G,H)**.

To effectively guide clinical treatment, we constructed a risk score model to stratify patients with PCa based on these nine RBP genes. The risk score for each PCa patient was calculated using the gene expressions levels multiplied by their coefficients from the multivariate Cox regression analysis. The detailed formula is as follows: Risk score = (0.31297 × *EXO1*) + (0.26564 × *PABPC1L*) + (0.32104 × *REXO2*) + (−0.05792 × *MBNL2*) + (0.15083 × *MSI1*) + (0.10192 × *CTU1*) + (−0.07827 × *MAEL*) + (−0.09089 × *YBX2*) + (−0.52454 × *ESRP2*). The patients were divided into high- and low-risk groups based on the median value of the risk score. The high-risk patients tended to have a worse prognosis compared with the low-risk patients (Figure 7A). Furthermore, the ROC curves showed a good performance of the model (Figure 7B); the AUC was 0.786 at 1 year, 0.758 at 3 years, 0.768 at 3 years, and 0.752 at 5 years. The model was further validated in GSE54460 (Figures 7C,D), GSE70768 (Figures 7E,F), and GSE70769 (Figures 7G,H). The high-risk patients in GSE70769 showed worse prognosis compared with the low-risk patients (*P*-value <0.05). Moreover, high-risk patients in GSE54460 and GSE70768 (Figures 7C,E, respectively) had a trend of worse prognosis compared with the low-risk patients despite a *P*-value >0.05. These results show the reliability and stability of the model in stratifying the patients. Moreover, the ROC curves showed a good performance in GSE54460 (Figure 7D) and GSE70769 (Figure 7H) with all AUCs larger than 0.6. The AUC for GSE70768 was 0.763 at 1 year, showing good performance. However, AUCs for time larger than 2 years were relatively low.

### Associations Between the Model and Clinicopathological Factors of PCa

**FIGURE 8:**
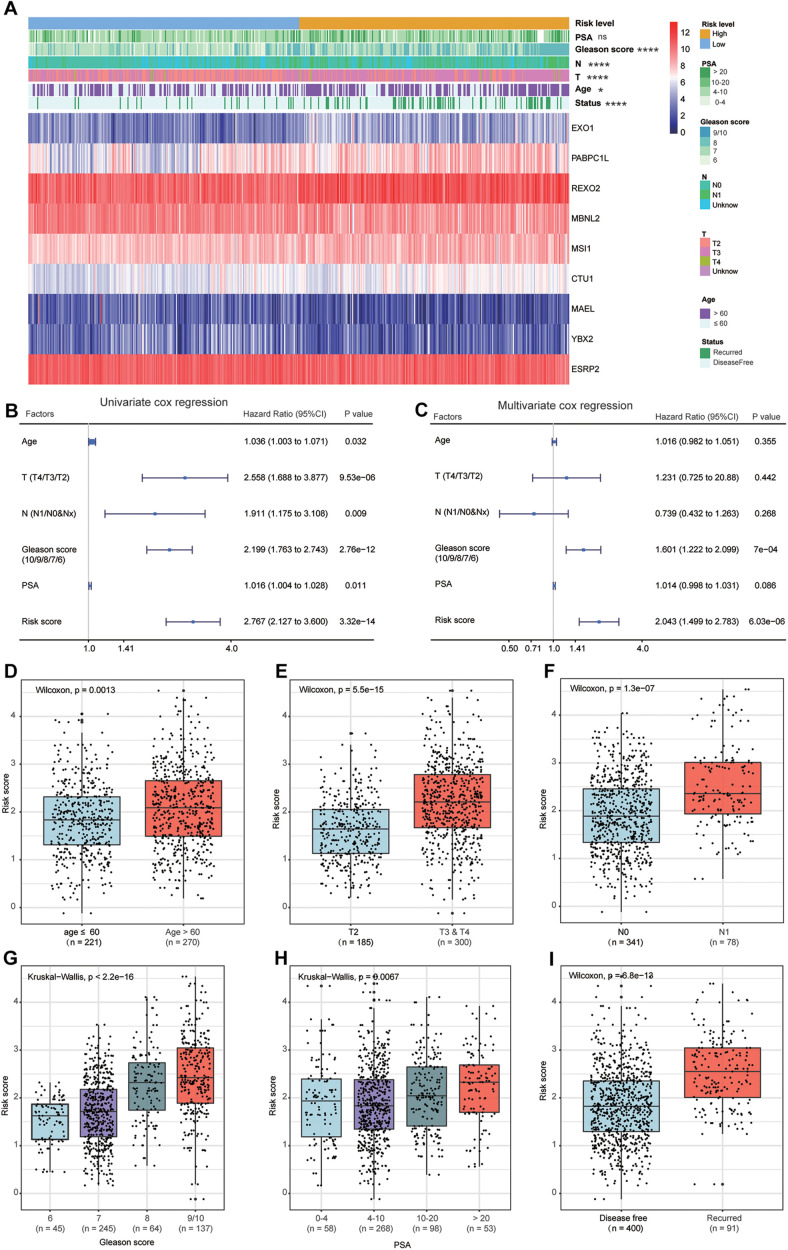
Relationship between the clinical factors and risk score model. **(A)** The heat map shows the expression levels of nine genes in high-risk and low-risk patients. The clinical factors are compared between these two patient groups, showing that high-risk patients have higher proportions of high Gleason score, lymph node metastasis, high pathological T staging, advanced age, and recurrence rate. The univariate **(B)** and multivariate **(C)** Cox regression analyses for evaluating the relationship between the risk score model and clinical factors. The bar chart shows that patients with advanced age **(D)**, high T staging **(E)**, node metastasis **(F)**, high Gleason score **(G)**, high prostate-specific antigen **(H)**, and recurrence **(I)** tend to have a higher risk score. ns: *P* > 0.05, *P < 0.05, **P < 0.01, ***P < 0.001, ****P < 0.0001.

The heat map shows the expression levels of the nine RBPs and the distributions of the clinicopathological factors between the high- and low-risk patients (Figure 8A). The results showed that the high-risk patients had higher proportions of high Gleason score (*P* < 0.0001), lymph node metastasis (*P* < 0.0001), high pathological T staging (*P* < 0.0001), advanced age (*P* < 0.05), and recurrence rate (*P* < 0.0001). The detailed distribution of the clinicopathological data across patient subgroups were shown in Table 1. The univariate Cox regression analysis showed that the risk score model was a risk factor for disease-free survival in PCa patients (Figure 8B), and the multiple Cox regression analysis revealed that the risk score model was an independent risk factor for disease-free survival after integration with age, pathological T staging, lymph node status, Gleason score, and PSA level (Figure 8C). In addition, we compared the risk scores between different clinical subgroups and found that patients with advanced age (Figure 8D), high pathological T staging (Figure 8E), lymph node metastasis (Figure 8F), high Gleason score (Figure 8G), high PSA (Figure 8H), and recurrence status (Figure 8I) tended to have higher risk scores. These results demonstrated that our risk score model was closely correlated with the clinicopathological factors of PCa.

**TABLE 1:** Association between the risk score model and patients’ clinical characteristics.

Variables	TCGA cohort (*n* = 491), *n* (%)	Risk score		*P* value
			
		Low risk	High risk	
Age (mean ± SD, years)	61.0 ± 6.8	60.2 ± 7.0	61.7 ± 6.6	0.013
≤60 years	221 (45.0)	126 (51.2)	95 (38.8)	0.007
>60 years	270 (55.0)	120 (48.8)	150 (61.2)	
Pathological T stage				<0.001
T2	185 (37.7)	128 (52.1)	57 (23.3)	
T3	290 (59.1)	110 (44.7)	180 (73.4)	
T4	10 (2.0)	4 (1.6)	6 (2.5)	
Tx	6 (1.2)	4 (1.6)	2 (0.8)	
Nodal stage				<0.001
N0	341 (69.4)	179 (72.8)	162 (66.1)	
N1	78 (15.9)	20 (8.1)	58 (23.7)	
Nx	72 (14.7)	47 (19.1)	25 (10.2)	
Gleason score				<0.001
6	45 (9.2)	35 (14.2)	10 (4.1)	
7	245 (49.9)	153 (62.2)	92 (37.5)	
8	64 (13.0)	21 (8.6)	43 (17.6)	
9	134 (27.3)	36 (14.6)	98 (40.0)	
10	3 (0.6)	1 (0.4)	2 (0.8)	
PSA at initial diagnosis (ng/ml)				0.004
≤4	58 (11.8)	29 (11.8)	29 (11.8)	
(4,10]	268 (54.6)	153 (62.2)	115 (46.9)	
(10,20]	98 (19.9)	41 (16.7)	57 (23.3)	
>20	53 (10.8)	17 (6.9)	36 (14.7)	
Unknown	14 (2.9)	6 (2.4)	8 (3.3)	

**FIGURE 9:**
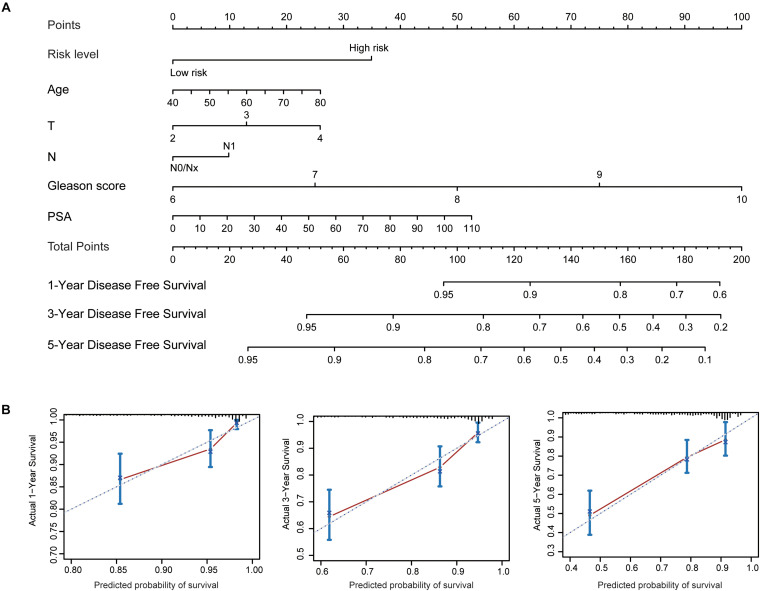
Established nomogram and calibration plots for predicting patients’ disease-free survival with prostate cancer. **(A)** Nomogram integrating the risk score model and clinical factors for prediction of 1-, 3-, and 5-year disease-free survival. **(B)** Calibration plots showing high predictive accuracy of the nomogram.

To better predict patients’ prognosis and guide clinical practice, we integrated the risk score model and clinical factors of PCa to construct a nomogram (Figure 9A). The clinical factors included were risk factors for disease-free survival of PCa patients. Calibration plots were used to evaluate the performance of nomogram (Figure 9B) and showed good performance for predicting 1-, 3-, and 5-year disease-free survival probabilities. Moreover, we calculated Harrell’s concordance index (C-index) to evaluate the powers of selected factors (Table 2). As the results showed, the risk score model had a relative higher C-index [0.659; 95% confidence interval (CI): 0.610–0.708]. Further, the combination of the risk score model with clinical factors has a higher C-index (0.741; 95% CI: 0.684–0.798) than the risk score model or clinical factors alone, suggesting that combining the risk score model with clinical factors could improve prognostic accuracy for PCa patients.

**TABLE 2:** Comparison of the predictive powers of multiple factors in TCGA dataset.

Factors	Disease free survival	
	C-index	95% CI
Age	0.579	0.522–0.636
Pathological T stage	0.614	0.565–0.663
Nodal stage	0.540	0.491–0.589
Gleason score	0.699	0.642–0.756
PSA	0.558	0.491–0.625
Risk level	0.659	0.610–0.708
Risk level + Age + Pathological T stage + Nodal stage + Gleason score + PSA	0.741	0.684–0.798

*Abbreviations: C–index, Harrell’s concordance index; CI, confidence interval.*

## Discussion

PCa is the second most common cancer in men and poses a growing burden on healthcare systems worldwide (Mistry et al., 2011). It was estimated that almost 1.3 million new cases of PCa were diagnosed and that 359,000 associated deaths occurred worldwide in 2018 (Bray et al., 2018). Currently, the conventional treatment modalities for PCa include surgical resection, hormone therapy, radiotherapy, and chemotherapy (Abou et al., 2020). Moreover, the dichotomy of overtreatment and tumor progression of PCa poses a new challenge for modern medicine, owing to the substantial heterogeneity of PCa. Therefore, the exploration of molecular biomarkers and construction of an excellent risk stratification model for PCa patients will be useful for predicting the degree of malignancy and prognosis and guiding clinical treatments. Further, high-throughput sequencing and bioinformatics technologies provide convenient and effective tools to identify pivotal biomarkers for PCa and uncover their molecular functions (Bass et al., 2014). Prior studies have revealed that RBPs play vital roles in tumorigenesis and progression of PCa by regulating multiple fundamental biological processes through posttranscriptional events (Gerstberger et al., 2014b; Harvey et al., 2017). As the molecular functions of most RBPs in PCa remain unclear, we systematically investigated them in our present study. We obtained 59 differentially expressed RBPs, comprising 28 upregulated and 31 downregulated RBPs; subsequently, we explored the potential functional pathways and constructed a PPI network for the differentially expressed RBPs. The expression levels, genomic mutations, and prognostic and diagnostic values of the key RBPs were comprehensively assessed. Moreover, we implemented consensus clustering to determine the overall roles of these genes in PCa. Finally, we constructed a risk score model based on nine survival-related RBPs to predict the prognosis of the PCa patients and validated the efficiency of the model in three external datasets.

Studies have indicated that RNA splicing widely participates in posttranscriptional gene regulation and plays vital roles in the tumorigenesis and progression of cancer (De Maio et al., 2018). Further, RBPs are a critical factor and major component of the splicing machinery (Han et al., 2013); for example, HNRNPL drives the progression of PCa by directly regulating the targeted RNA alternative splicing (Fei et al., 2017). Meanwhile, RBPs also maintain the stability of various target RNAs to inhibit tumorigenesis and metastasis of multiple tumors, such as hepatocellular carcinoma (Han et al., 2019), breast cancer (Cheng et al., 2017), and glioblastoma (Vo et al., 2012). Moreover, the RBPs regulate biological processes at the posttranscriptional level and can function as activators or suppressors to affect tumor development and progression (Hopkins et al., 2016; Han et al., 2019; Iino et al., 2020) through multiple biological pathways. For example, NELFE could promote the progression of hepatocellular carcinoma by regulating MYC signaling (Dang et al., 2017), and TTP could inhibit cell proliferation and accelerate cell death in lung cancer through the autophagy pathway (Dong et al., 2018). However, the biological functions of most RBPs remain unexplored. In our study, the GO enriched terms showed that the differentially expressed RBPs were mainly enriched in translation, rRNA processing, mRNA processing, RNA splicing, nucleolus, cytosolic large ribosomal subunit, cytoplasm, RNA binding, poly(A) RNA binding, nucleic acid binding, and nucleotide binding, while the KEGG pathway analysis indicated that the upregulated RBPs in PCa could influence the occurrence and progression of cancer by regulating various pathways of ribosome, mRNA surveillance, RNA degradation, and RNA transport. Moreover, the key module identified from the PPI network revealed that the biological functions of module 1 were mainly involved in cytosolic large ribosomal subunits and polysomal ribosomes. As the differentially expressed RBPs were involved in multiple functional pathways and biological processes, it indicates their pivotal role in the occurrence and development of PCa.

In the present study, we identified nine survival-related RBPs: EXO1, PABPC1L, REXO2, MBNL2, MSI1, CTU1, MAEL, YBX2, and ESRP2. The Kaplan–Meier curves further confirmed their prognostic values; moreover, the associations with pathological T staging, pathological grade, and Gleason score of these nine RBPs were comprehensively evaluated. We found high expression levels of EXO1 and REXO2 and low expression levels of YBX2 and ESRP2 in samples with high pathological T staging, high pathological grade, and high Gleason score. Among these nine RBPs, the expression level of EXO1 was significantly correlated with clinical progression and prognosis of PCa. Patients with a high expression level of EXO1 showed poor prognosis and a high risk of lymph node metastasis (Luo et al., 2019). Moreover, ESRP2 is also overexpressed in PCa and is involved in AR-mediated splicing patterns (Munkley et al., 2019). However, the roles of other RBPs have not been reported in PCa but have been implicated in other cancers. For instance, PABPC1L is highly expressed in colorectal cancer and is significantly correlated with its clinical stage and prognosis (Wu et al., 2019). REXO2 has a 3′-to-5′ exonuclease activity, and its dysregulation leads to tumorigenesis of pheochromocytoma by disturbing the DNA replication, recombination, and repair processes (Laitman et al., 2020). MBNL2 possesses antitumor activity in lung and breast cancers and can inhibit cancer cell metastasis via the pAKT/EMT signaling pathway (Zhang et al., 2019). MSI1 regulates the Wnt and Notch signaling pathways; small molecule inhibitors targeting MSI1 have been investigated as blockers of cancer cell growth (Lan et al., 2015). CTU1 is crucial for maintaining genome stabilization and integrity, and its dysregulation can result in defects in the translation processes (Dewez et al., 2008). Finally, MAEL plays a key oncogenic role in bladder cancer by downregulating MTSS1 (Li et al., 2016). In the present study, these nine RBP genes showed a moderate diagnostic efficiency in differentiating PCa from normal samples. Hence, these RBP genes may be used as diagnostic and prognostic biomarkers for PCa in the future.

Two PCa subgroups (clusters 1 and 2) were identified after a consensus clustering analysis. Then, PCA confirmed the reliability of the two subgroups, and Kaplan–Meier curves showed significantly different prognoses between them. The patients in cluster 2 tended to have a worse prognosis and were associated with several oncogenic pathways involving E2F targets, G2M checkpoint, protein secretion, and mTORC1 signaling. These pathways are involved in the occurrence and progression of tumors; for instance, many cancer cells have defective G1 checkpoint mechanisms and thus depend upon the G2M checkpoint more than normal cells (Schmidt et al., 2017). Further, it is well known that mTORC1 signaling is necessary for cellular growth and metabolism and that its dysregulation is closely related to various human diseases, including cancers (Thomas et al., 2016; Ben-Sahra and Manning, 2017; Hare and Harvey, 2017). Therefore, a systematic exploration of the roles of these oncogenic pathways in PCa and their relationships with RBPs might provide novel insights for the treatment of PCa in the future.

Along with the advent of precision cancer medicine, more specific and effective risk stratification models are urgently needed to guide clinical practice and further improve the prognosis of PCa patients. In recent years, a variety of stratification models for PCa have been proposed; for example, Brockman et al. (2015) validated a model to predict the long-term risk of death of PCa patients with biochemical recurrence after undergoing surgical resection. Further, Van Neste et al. (2016) developed a multimodal risk model to identify high-grade PCa based on urinary molecular biomarkers and clinical risk factors, thus decreasing overtreatment. Mehralivand et al. (2018) constructed a risk model based on magnetic resonance imaging and clinical parameters to improve the predictive accuracy of PCa. In addition, Thurtle et al. (2019) introduced an individual multivariable predictive model that allowed the evaluation of potential treatment benefits for the PCa patients. Although these models showed good performance in predicting the therapeutic response or prognosis of PCa, some defects still exist as PCa is associated with complicated and polyfactorial tumors. Therefore, a single biomarker might have a limited effect on PCa prognosis (Jadvar, 2011). Hence, after considering the critical role of RBPs in the oncogenesis and progression of PCa, we constructed a risk score model based on nine survival-related RBPs for the prognostic stratification of the PCa patients. To our knowledge, this is the first PCa risk score model based on multi-RBPs and could be used to improve the evaluation of PCa patient prognosis. Our model showed significantly different prognoses for the high- and low-risk patients. Additionally, the model was validated using three external datasets (GSE54460, GSE70768, and GSE70769), and all three external datasets showed worse prognosis in the high-risk patients. We also investigated the correlations between the model and clinical factors. The results revealed that the high-risk PCa patients tended to have advanced stage, high Gleason score, high ratio of lymph node metastasis and recurrence, and poor prognosis, suggesting that our model was closely associated with traditional clinical variables. In addition, we found that this model was an independent risk factor for predicting disease-free survival in PCa patients. In general, our risk model shows great clinical applicability in distinguishing high-risk PCa patients and may be beneficial for early interventions to improve the clinical therapeutic effect.

Inevitably, our risk score model also has several limitations. All data used in the present study were obtained from public databases. Hence, a prospective study to further validate the efficacy of our model is needed. Moreover, the detailed functions and potential mechanisms of these nine RBP genes in PCa need to be further explored.

## Conclusion

Our study systematically explored the potential roles of RBPs in PCa and identified nine survival-related differentially expressed RBP genes. The expression levels of these RBPs were validated in the HPA database, and their associations with clinical traits were evaluated. All nine RBPs showed good diagnostic and prognostic values for PCa. Moreover, the risk score model based on these nine RBP genes could stratify PCa patients into two subgroups with different prognoses and malignant phenotypes and showed high associations with the clinical traits of PCa. Thus, we believe that our risk score model could improve the evaluation of treatment response and prognosis in PCa patients.

## Data Availability Statement

The datasets presented in this study can be found in online repositories. The names of the repository/repositories and accession number(s) can be found in the article/Supplementary Material.

## Author Contributions

CL and ST conceived and designed the experiments. XH acquired and analyzed the data. SG wrote the manuscript. JC analyzed the data. LZ checked the manuscript. All authors read and approved the final manuscript.

## Conflict of Interest

The authors declare that the research was conducted in the absence of any commercial or financial relationships that could be construed as a potential conflict of interest.

## Footnotes

1 https://portal.gdc.cancer.gov/

2 https://www.ncbi.nlm.nih.gov/geo/

3 https://xenabrowser.net/

4 http://cbioportal.org

5 http://www.bioconductor.org/packages/release/bioc/html/limma.html

6 https://cran.r-project.org/web/packages/rsem/index.html

7 https://david.ncifcrf.gov/

8 https://string$-$db.org/

9 http://cytoscape.org/

10 https://cran.r-project.org/web/packages/survival/index.html

11 https://cran.r-project.org/web/packages/pROC/index.html

12 http://www.bioconductor.org/packages/release/bioc/html/ConsensusClusterPlus.html

## Supplementary Material

Supplementary Figure 1 Selection of the most valuable RNA binding proteins for the prostate cancer patients. **(A)** The coefficients of the least absolute shrinkage and selection operator Cox regression analysis, used to identify the most valuable prognostic genes, are shown. **(B)** Plot of partial likelihood deviance for eighteen genes in TCGA dataset.

Supplementary Figure 2 Evaluation of the diagnostic values of the nine RNA binding proteins between the prostate cancer and normal samples: **(A)** EXO1; **(B)** PABPC1L; **(C)** REXO2; **(D)** MBNL2; **(E)** MSI1; **(F)** CTU1; **(G)** MAEL; **(H)** YBX2; **(I)** ESRPS. **(J)** The mutation and copy number alterations for the nine RNA binding proteins.

Supplementary Figure 3 Identification of cluster numbers using consensus clustering. The consensus clustering matrix for *k* = 2 **(A)**; *k* = 3 **(C)**; *k* = 4 **(E)**; and *k* = 5 **(G)**. A principal component analysis was used to evaluate the distribution of different cluster numbers. **(B)** Two clusters; **(D)** three clusters; **(F)** four clusters; **(H)** and five clusters.

Supplementary Table 1 The sheet of differentially expressed RNA binding proteins for prostate cancer in TCGA dataset.

Supplementary Table 2 The results of functional enrichment analysis for upregulated differentially expressed RNA binding proteins.

Supplementary Table 3 The results of functional enrichment analysis for downregulated differentially expressed RNA binding proteins.

Supplementary Table 4 The results of univariate Cox regression analysis to select survival-related RNA binding proteins.

## Abbreviations

ADT: androgen deprivation therapy
AR: androgen receptor
AUC: area under the curve
CAN: copy number alteration
CRPC: castration-resistant prostate cancer
GEO: Gene Expression Omnibus
GO: gene ontology
GSEA: Gene Set Enrichment Analysis
HPA: The Human Protein Atlas
KEGG: Kyoto Encyclopedia of Genes and Genomes
MCODE: Molecular Complex Detection
NES: normalized enrichment score
PCA: principal component analysis
PCa: prostate cancer
PPI: protein–protein interaction
PSA: prostate-specific antigen
RBP: RNA binding protein
ROC: receiver operating characteristic
TCGA: The Cancer Genome Atlas.
