# Supplementary figures and images for: Effects of RNA Binding Proteins on the Prognosis and Malignant Progression in Prostate Cancer
Source: Front Genet. 2020 Oct 20;11:591667. doi: 10.3389/fgene.2020.591667 (PMC7606971; doi:10.3389/fgene.2020.591667)

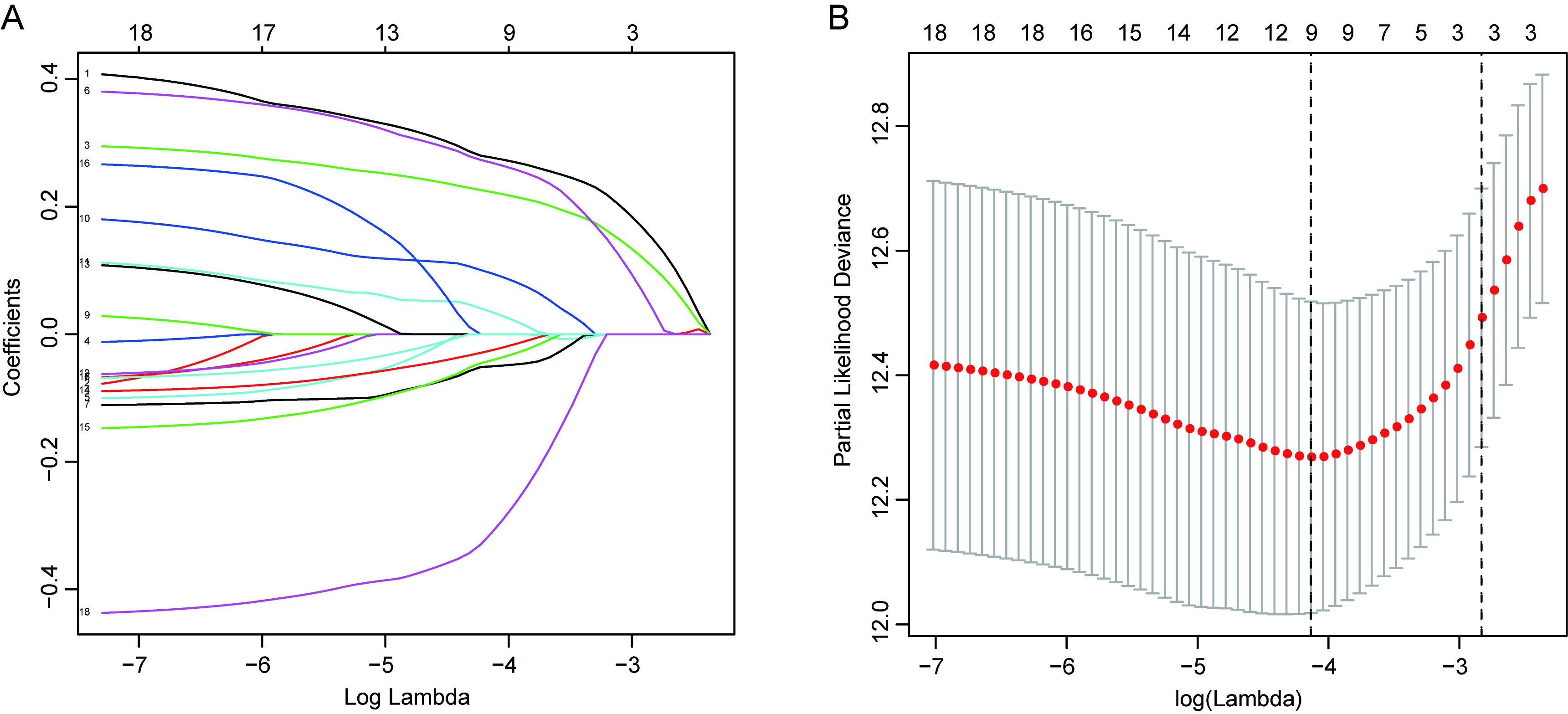

Supplement: Supplementary Figure 1 — Selection of the most valuable RNA binding proteins for the prostate cancer patients. (A) The coefficients of the least absolute shrinkage and selection operator Cox regression analysis, used to identify the most valuable prognostic genes, are shown. (B) Plot of partial likelihood deviance for eighteen genes in TCGA dataset. [file Image_1.TIF]

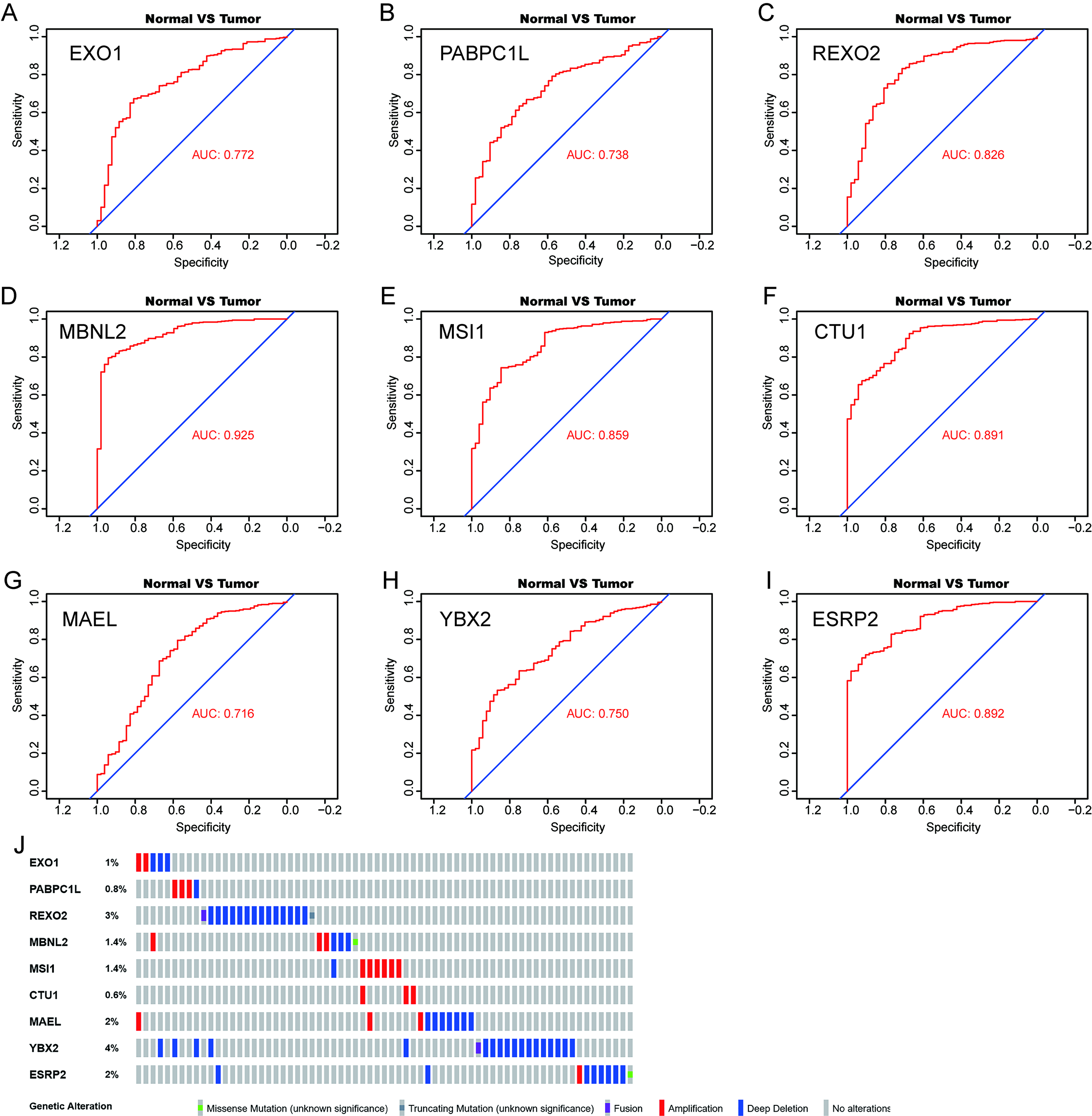

Supplement: Supplementary Figure 2 — Evaluation of the diagnostic values of the nine RNA binding proteins between the prostate cancer and normal samples: (A) EXO1; (B) PABPC1L; (C) REXO2; (D) MBNL2; (E) MSI1; (F) CTU1; (G) MAEL; (H) YBX2; (I) ESRPS. (J) The mutation and copy number alterations for the nine RNA binding proteins. [file Image_2.TIF]

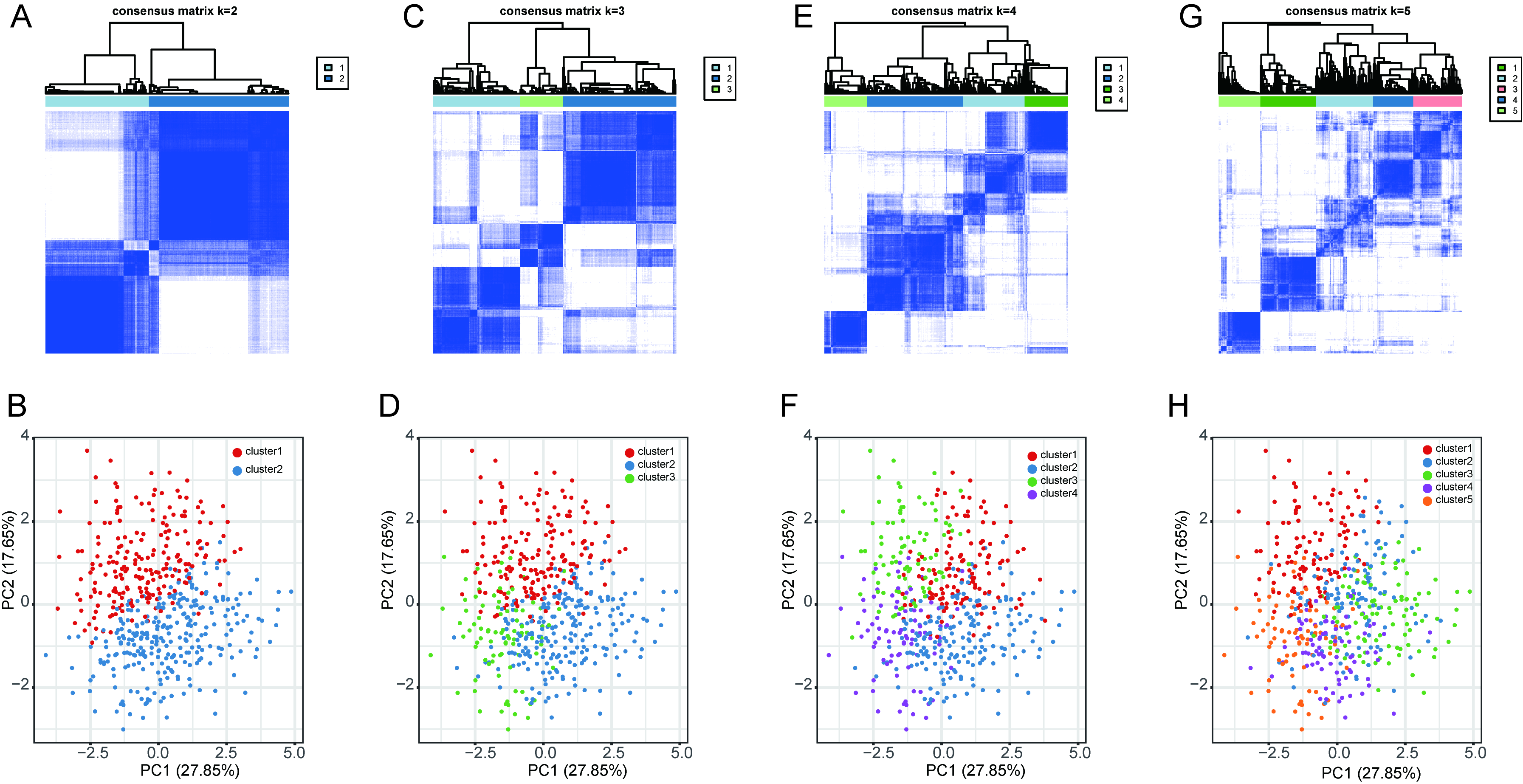

Supplement: Supplementary Figure 3 — Identification of cluster numbers using consensus clustering. The consensus clustering matrix for k = 2 (A); k = 3 (C); k = 4 (E); and k = 5 (G). A principal component analysis was used to evaluate the distribution of different cluster numbers. (B) Two clusters; (D) three clusters; (F) four clusters; (H) and five clusters. [file Image_3.TIF]
